# Structural Evolution in Isothermal Crystallization Process of Poly(L-lactic acid) Enhanced by Silk Fibroin Nano-Disc

**DOI:** 10.3390/ma12111872

**Published:** 2019-06-10

**Authors:** Amit Kumar Pandey, Vimal Katiyar, Hideaki Takagi, Nobutaka Shimizu, Noriyuki Igarashi, Sono Sasaki, Shinichi Sakurai

**Affiliations:** 1 Department of Biobased Materials Science, Kyoto Institute of Technology, Kyoto 606-8585, Japan; amitpandey886@gmail.com (A.K.P.); sono@kit.ac.jp (S.S.); 2 Department of Chemical Engineering, Indian Institute of Technology Guwahati, Guwahati, Assam 781039, India; vkatiyar@iitg.ac.in; 3 High Energy Accelerator Research Organization (KEK), Tsukuba 305-0801, Japan; takagih@post.kek.jp (H.T.); nobutaka.shimizu@kek.jp (N.S.); noriyuki.igarashi@kek.jp (N.I.)

**Keywords:** poly(L-lactic acid), silk fibroin nano-disc, crystalline lamella, isothermal crystallization, lamellar stacking, small-and wide-angle X-ray scattering simultaneous measurement

## Abstract

The nucleating effect of silk fibroin nano-disc (SFN) on the crystallization behavior of poly(L-lactic acid) (PLLA) was investigated by simultaneous synchrotron small- and wide-angle X-ray scattering measurements. For the isothermal crystallization at 110 °C from the melt, the induction period of the PLLA specimens containing 1% SFN was reduced compared to that of the neat specimens, indicating the acceleration of the nucleation of PLLA. The final degree of crystallinity was also increased, and the crystallization half-time was decreased, which indicates that the overall crystallization process was accelerated. Furthermore, the final value of the crystallite size (the lateral size of the crystalline lamella) was slightly lower for the specimens containing 1% SFN than that for the PLLA neat specimen, although the crystallites started growing much earlier. However, it was found that there was no effect of SFN on the growth rate of the crystallite size. The lamellar thickening process was also accelerated with a clear overshooting phenomenon with the inclusion of 1% SFN. As for the polymorphism, the α’ phase is dominant with about 96%, but a small amount of the α phase (4%) is found to exist. It was found that the SFN can also accelerate the formation of the minor α phase as well as the major α’ phase.

## 1. Introduction

The study of the formation of the crystalline structure during the crystallization process has been attracting a lot of interest of researchers to develop better physical properties, especially for poly(L-lactic acid) (PLLA), which is one of the most popular biobased polymers. PLLA is synthesized from the monomer, L-lactic acid, either by condensation or ring opening polymerization. L-lactic acid is obtained from 100% renewable resources like corn and sugarcane. Over recent decades, PLLA has become an exciting material as a possible replacement to the conventional polymers. Although the modulus and ultimate strength of PLLA are comparable to poly(ethylene terephthalate), the industrial applications of PLLA are limited because of its slow crystallization rate and a low degree of crystallinity. Understanding and controlling of the crystallization process are of essential importance in the processing of PLLA as its thermal and mechanical properties are dependent on the crystallization speed, crystallite size and crystallinity. Therefore, it is important to clearly understand the relationship between the crystalline structure and the properties. 

The relationship between polymorphism (the ability of a solid material to exist in more than one form) and mechanical properties of polymers has long been an interesting subject. A well-known example of the polymers exhibiting polymorphism is isotactic polypropylene (iPP), which shows three different crystal modifications (α, β, and γ) depending on various conditions [1]. The β-form of iPP is widely popular in industrial applications as it exhibits significantly improved toughness and impact resistance compared to the usual α-form [2]. Another example of polymorphism is for a biobased polymer, poly(3-hydroxybutyrate) (P3HB). The P3HB can crystallize in two forms α and β depending on the crystallization conditions. It is known that the β-form shows higher strength and modulus compared to the α-form [2]. The transition of the polymorphic structures during processing also affects the mechanical performance of a polymer.

The crystalline structure of PLLA has already been studied by many researchers [3,4,5,6,7]. It has been reported that the crystallization of PLLA leads to several crystal forms (α, α’, β, and γ) [5]. The α form is the most stable polymorph that is developed from the melt or solution [8]. The β form is formed by stretching the α form at high temperature and high drawing ratio, whereas the γ form is formed through the epitaxial crystallization of PLLA on hexamethylbenzene substrate [8,9]. Among the crystal forms, α and α’ are the common crystalline structures as the β and γ forms can only be developed under the special conditions. Both of the α and α’ crystals have left-handed 10_3_ helical conformation of PLLA chains, while the α’ crystal is less ordered as compared to the α crystal. It has been reported that the α’ crystal has slightly larger values of the edges of the crystalline lattice than those of the α phase [5]. Due to their slightly looser packing and disordered structure, the α’ crystals show less brittleness, higher impact strength and higher elongation at break compared to the α crystals [8]. The fraction of the α and α’ crystals formed in the isothermal crystallization from the melt is strongly dependent on the crystallization temperature. According to reference [5], the formation of α’ phase only occurs when the crystallization temperature is lower than 120˚C. It has also been reported that α’-to-α crystal transition takes place during heating [5]. This transition mainly involves the slight rearrangement of molecular packing within the unit cell in order to accommodate the more energy-favorable state, corresponding to the reduction of lattice dimensions [5].

As mentioned above, the crystallization of PLLA is very slow, which limits its wide applications. The addition of a nucleating agent is one of the effective methods to accelerate the crystallization of PLLA. Most of the nucleating agents reported for PLLA (talc, carbon nanotubes, clay) are inorganic materials, which are non-biodegradable in nature. Recently, it is a growing trend to use natural resources like cellulose and starch as a nucleating agent for PLLA [8]. In this regard, we used silk fibroin nano-disc (SFN) as a crystallization promoter for PLLA. The SFN is a biobased and environmentally benign material which is extracted from the waste of muga silk cocoons [10]. The nucleating effect of the SFN for PLLA is reported in our previous study [11], where we carried out the differential scanning calorimetry (DSC) measurements and polarizing optical microscope (POM) observations to understand the crystallization behavior of PLLA in the presence of 1% SFN. We found that the addition of 1% SFN accelerated nucleation of PLLA, while the spherulite growth rate was not accelerated by SFN. It is well-known that the crystallizability of PLLA is strongly dependent on its optical purity. As the D moiety in PLLA increases, the crystallization rate decreases. Therefore, the effect of D moiety in PLLA on the acceleration ability of SFN is also addressed in our previous and the current study.

In the current study, the time-resolved synchrotron wide-angle X-ray scattering (WAXS) and small-angle X-ray scattering (SAXS) techniques are employed to study the evolution of the structure and the kinetics of the crystallization. The fraction of α and α’ phases, crystallite size (lateral size of crystalline lamellae), long period and thickening of the lamella during the isothermal crystallization process are analyzed, and changes in these structural parameters as a function of time during the isothermal crystallization upon T-jump from 200 °C to 110 °C are examined in order to discuss the effects of the SFN loading. 

## 2. Experimental

### 2.1. Specimens

The PLLA samples used in this study were obtained from NatureWorks, Minnetonka, MN, USA. The sample characteristics are summarized in Table 1. The SFN is a nanoparticle that was extracted from wastes of the muga silk (*Antheraea assama*) cocoon [10]. The crystalline portion (the β sheets) of the silk fibroin was isolated by using the acid-hydrolysis method. The obtained extract comprises 83.8% L-alanine, and the well-defined disc-like nano particles were obtained. Such morphology and dimensions have been reported in the previous paper [10] as the average diameter and thickness of ~45 nm and ~3 nm, respectively. The detailed information about the SFN can be found in reference [10]. The composites of PLLA and SFN were prepared by the solution-casting method by using dichloromethane (DCM) as a solvent. The details of the specimens’ preparation has been reported in our previous study [11]. The specimens are labeled as D1.4/SFN(x) or D0.5/SFN(x), where the numbers after *D* denote the % of D moiety in PLLA, and *x* stands for % of SFN inclusion.

### 2.2. Simultaneous Small- and Wide-Angle X-ray Scattering (SWAXS) Measurements

The time-resolved SWAXS measurements were carried out by using the synchrotron radiation as an X-ray source at the beamline BL-6A of Photon Factory at the KEK (High-Energy Accelerator Research Organization) in Tsukuba, Japan. The wavelength of the incident X-ray beam was 0.150 nm. The sample was packed into an aluminum cell, which had a diameter of 4 mm and thickness of 1 mm. The specimens were sandwiched by a couple of pieces of the polyimide (Kapton) film, obtained from DuPont-Toray Co., Ltd., Tokyo, Japan. Firstly, the specimens were melted at 200 °C for approximately 5 min, and then the sample cell was quickly moved to another heater block, which was maintained at 110 °C, and then the simultaneous SWAXS measurement was performed. Note here that this isothermal crystallization temperature (110 °C) can be considered to be located in regime III. This is because Song et al. (2018) have reported that PLLA specimens with 100% and 98% L moieties show the regime II at higher temperature and regime III at lower temperature with the transition temperature of 120 °C [12]. The actual sample temperature was monitored by a temperature sensor, which was directly attached to the sample. The stabilization time for the temperature equilibration after the jump was 15 s and the temperature fluctuation was less than 0.3 °C. More details of the experimental setup of the SWAXS measurement are reported elsewhere [13]. The scattering vector *q* was calibrated by using collagen for SAXS and polyethylene for WAXS. The SAXS and WAXS patterns were recorded at every 5 s with the X-ray exposure time of 5 s. The background scattering was subtracted. The one-dimensional SAXS and WAXS profiles were obtained by taking the circular average of the 2d-SAXS pattern and the sector average of the 2d-WAXS pattern, respectively.

## 3. Results and Discussion

### 3.1. WAXS Results

**Figure 1:**
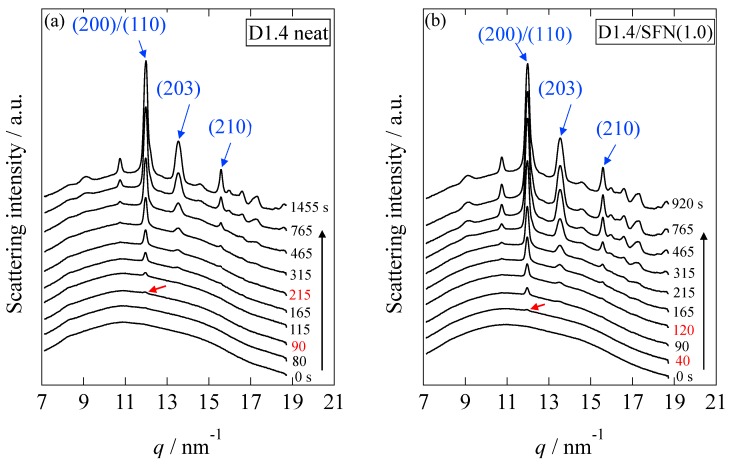
Time-resolved wide-angle X-ray scattering (WAXS) profiles of (**a**) D1.4 neat (**b**) D1.4/SFN(1.0) specimens. The red arrow indicates the first detection of the peak.

The changes in the WAXS profiles were measured in the isothermal crystallization process at 110 °C from the melt (200 °C). Figure 1 shows the WAXS profiles for the D1.4 neat and D1.4/SFN(1.0) specimens as a function of time. Here, *q* denotes the magnitude of the scattering vector, as defined by *q* = (4π/*λ*) sin(*θ*/2), with *λ* and *θ* being the wavelength of X-ray and the scattering angle, respectively. The indexing of the observed reflections is based on the crystal structure of PLLA reported in the references [3,4,5]. 

As shown in Figure 1, in the early stage, there is no crystalline peak, which shows the presence of 100% amorphous phase. As time goes on, a crystalline peak appears (which has been shown by the red arrow in Figure 1). The induction period (*t*_0_) of the crystallization is evaluated from the first detection of the crystalline peak. It can be seen from Figure 1 that the loading 1% SFN caused the reduction of the induction period from 90 s to 40 s, which shows the enhancement of the crystallizability by SFN. The time evolution of the degree of crystallinity was calculated from the WAXS profiles by using the following equation:

(1) $$\phi_{\mathrm{WAXS}}=\frac{\Sigma A_{c}}{\Sigma A_{c}+A_{a}}$$

**Figure 2:**
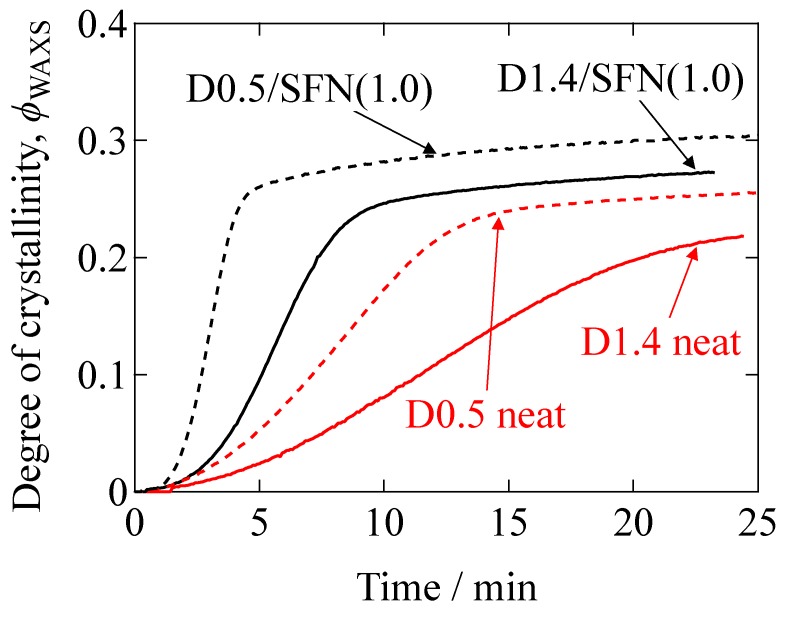
Degree of crystallinity as a function of time for the isothermal crystallization at 110 °C.

Here, *ΣA_c_* is the summation of the peak area of the crystalline peaks, and *A*_a_ is the peak area of the amorphous halo. The peak decomposition was conducted, and the degree of crystallinity (*ϕ*_WAXS_) was evaluated. The calculated *ϕ*_WAXS_ is plotted as function of time in Figure 2. As can be seen from Figure 2, the final degree of the crystallinity has been increased by the inclusion of 1% SFN. Judging from the slope of the curve at *t* = *t*_0.5_ (where *t*_0.5_ is the crystallization half-time at the 50% of the final crystallinity attained) in Figure 2, it can be considered that the crystallizabilty of PLLA is increased by adding 1% SFN. Table 2 summarizes the induction period, the final degree of crystallinity and the crystallization half-time of all the specimens, calculated from the results of Figure 2. It is clearly observed that *t*_0.5_ is also decreased by loading of SFN, indicating the acceleration of the crystallization rate.

**Figure 3:**
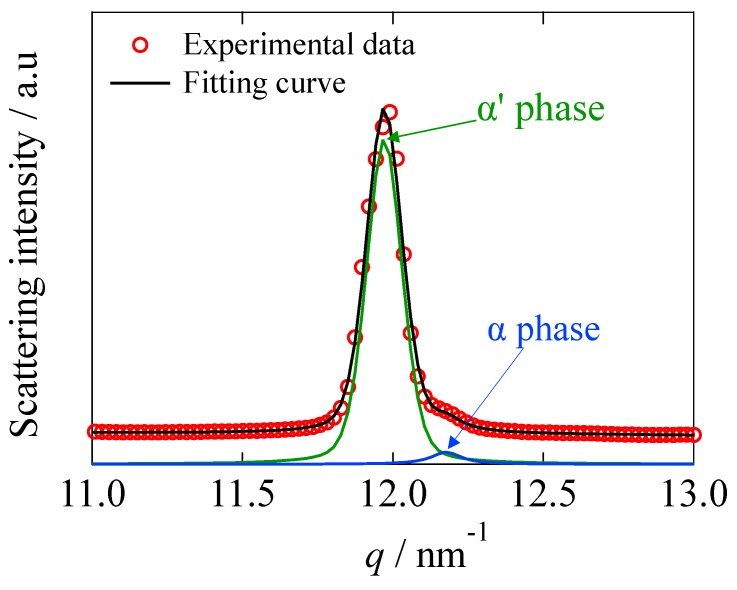
Decomposition of the (200)/(110) reflection peak in the range of 11 < *q* < 13 nm^−1^.

Furthermore, it is important to check the effect of SFN on the formation of the crystal polymorph (α and α’ phases). For this purpose, the evaluation of the fraction of α and α’ phases formed during the isothermal crystallization at 110 °C was conducted by analyzing the (200)/(110) reflection. Since there observed a small shoulder around *q* = 12.2 nm^−1^, we tried to decompose the shoulder peak (α phase) from the main peak (α’ phase). Figure 3 shows an example of the peak decomposition in the range of 11 < *q* < 13 nm^−1^, ensuring the perfect peak decomposition.

**Figure 4:**
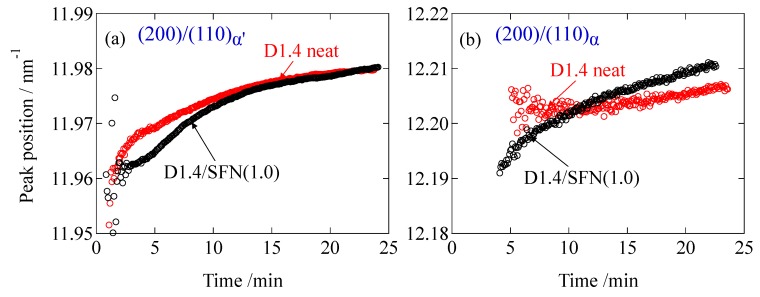
Changes in the position of the peak for (**a**) (200)/(110)_α’_ and (**b**) (200)/(110)_α_.

Figure 4 shows the changes in the peak positions of the α’ and α phases. The peak positions of both of the phases are continuously increasing as the crystallization process proceeds. These results show that loosely packed crystals are initially formed and gradually densified as a function of time. There was almost no effect of SFN on the overall behaviors of the changes in the peak position. 

On the basis of the area of the decomposed (200)/(110) peaks, the fraction of α and α’ crystals are evaluated as

(2) $$f_{\left(200\right)/{\left(110\right)}_{\alpha \;}}=\frac{A_{\left(200\right)/{\left(110\right)}_{\alpha \;}}}{A_{\left(200\right)/{\left(110\right)}_{\alpha^{\prime }\;}}+A_{\left(200\right)/{\left(110\right)}_{\alpha \;}}}$$

(3) $$f_{\left(200\right)/{\left(110\right)}_{\alpha^{\prime }\;}}=\frac{A_{\left(200\right)/{\left(110\right)}_{\alpha^{\prime }\;}}}{A_{\left(200\right)/{\left(110\right)}_{\alpha^{\prime }\;}}+A_{\left(200\right)/{\left(110\right)}_{\alpha \;}}}$$

where *A_x_* denotes the peak area of the decomposed *x* reflection.

**Figure 5:**
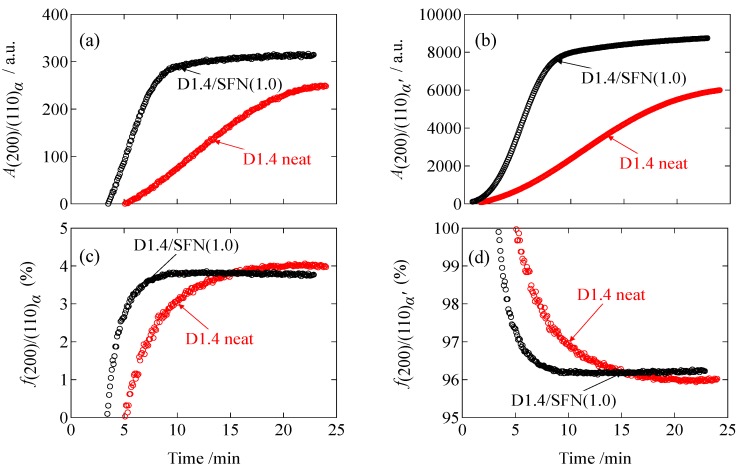
Plots of the peak area as a function of time for (**a**) the decomposed (200)/(110)_α_ peak and for (**b**) the decomposed (200)/(110)_α’_ peak, and fraction of (**c**) α phase and (**d**) α’ phase.

Figure 5a,b shows the peak area of the α and α’ phases as a function of time. Firstly, the α’ peak appeared, showing that loosely packed crystals are initially developed. After *t* = 5 min for the D1.4 neat or *t* = 3.5 min for the D1.4/SFN(1.0) specimen, the α phase starts appearing. The peak areas of both phases are increasing as a function of time. Figure 5c,d shows the fraction of the α and α’ phases evaluated by using Equations (2) and (3). As seen in Figure 5c, the fraction of the α phase is increasing in the early stage because of the successive formation of the α phase in the preceding α’ phase crystallites. However, the fraction of the α phase was leveled off at 3.8% to 4%. This means that the increasing behaviors for both phases are similar to each other, as can be seen in Figure 5a,b. Finally, it can be stated that the SFN may decrease the α fraction slightly. A similar result has been found for the case of loading a special plasticizer (organic acid monoglyceride; OMG) [13], where the reduction of the α phase is favorable because of the unfavorable nature of the lower impact strength of the α phase (more brittle than the α’ phase). Furthermore, we discuss whether the α phase is formed directly from the melt or due to the transition of the α’ phase. It has been reported [5] that the transition of α’-to-α phase takes place in heating prior to the melting. Since the temperature is kept constant (isothermal crystallization), it is needless to consider the α’-to-α crystal transition. Moreover, as shown in Figure 5b, the amount (peak area) of the α’ phase is not decreased even after the appearance of the α phase, which suggests that the α crystals are directly formed from melt instead of α’-to-α phase transition.

The average crystallite size (*D_hkl_*) in the direction normal to the (*hkl*) plane was evaluated by the Scherrer’s equation,

(4) $$D_{hkl}=\frac{K\lambda }{\beta_{hkl}\;\cos\left(\;\frac{\theta }{2}\;\right)}$$

where *K* is a constant (0.9) and *λ* is the wavelength of the incident X-ray. *β* is a full-width at half maximum (FWHM) in the unit of radian, and *θ* is the scattering angle.

**Figure 6:**
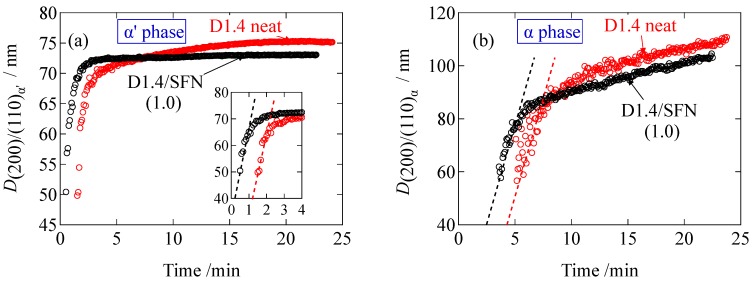
Comparison of the crystallite size evaluated by the Scherrer’s equation for (**a**) α’ phase and (**b**) α phase. Inset in (**a**) shows the expanded view.

As seen in Figure 6, the crystallite size is increasing as a function of time during the crystallization. The final value of the crystallite size for the D1.4/SFN(1.0) specimen is smaller than the D1.4 neat specimen. This result indicates that the presence of SFN decreases the final size of the crystallites of PLLA. The slope of the plots in Figure 6 can be considered as the crystallite growth rate. As can be seen in Figure 6a,b, the initial slope is almost constant, indicating that the SFN does not accelerate the growth of the crystallites. However, the SFN shortens the induction period (see also Figure 6a,b) as the evolution time is much shortened by SFN, suggesting that the SFN can enhance only the nucleation rate and not the crystallite growth rate. Similar results have been reported in our previous study for the spherulite growth based on the POM observation [11], where it was reported that the spherulite growth was unaffected by the inclusion of SFN, while the induction period was reduced. Finally, it should be noted that *D*_(200)/(110)_ stands for the lateral size of the lamellar crystallites. The initial slope of the plot in Figure 6a gives 30.0 nm/min of the growth rate of the lateral direction of the crystalline lamella (α’ phase), while the slope in Figure 6b gives the growth rate of 14.8 nm/min for the α phase. These values are much larger as compared to the growth rate of the lamellar thickness, which is 0.3 nm/min (as shown later). The much slower growth of the lamellar thickness is quite reasonable.

The kinetics of isothermal crystallization are described by the well-known Avrami theory [14,15,16], according to which the degree of crystallinity as a function of time [*ϕ*(*t*)] can be expressed as:

(5) $$\frac{\phi \left(t\right)}{\phi^{\infty }}=1-\exp\left[-k{\left(t-t_{0}\right)}^{n}\right]$$

(6) $$\log\left[-\ln\left(1-\frac{\phi \left(t\right)}{\phi^{\infty }}\right)\right]=n\;\log\left(k\right)+n\;\log\left(t-t_{0}\right)$$

**Figure 7:**
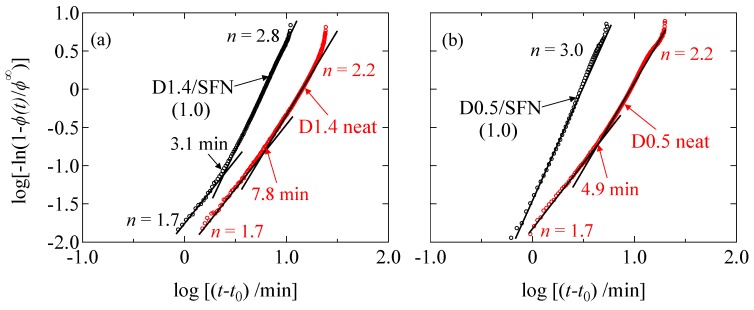
Avrami plots based on the WAXS results as shown in Figure 2 for (**a**) D1.4 specimens and (**b**) D0.5 specimens. Here, *t*_0_ denotes the induction period.

Here, *ϕ*^∞^ denotes the degree of crystallinity after the complete crystallization, *t*_0_ is the induction period for the crystallization, *n* is the Avrami exponent which represents the dimensionality of the growing crystallites and *k* is the crystallization rate constant, which contains the contributions from the nucleation and the growth of crystal. The Avrami plots for the D1.4 neat, D1.4/SFN(1.0), D0.5 neat and D0.5/SFN(1.0) specimens are shown in Figure 7. Here, the initial slope of the Avrami plots was different from that in the later stage, suggesting the change in the mode of the crystal growth. The crossover time is earlier for the D0.5 neat specimen than the D1.4 neat specimen. However, in the case of the D0.5/SFN(1.0) specimen, such crossover behavior was not observed. The similar tendency of the Avrami plots has been found in our previous study [11] in which the Avrami plots are made on the basis of the results of the DSC measurements. In the early stage of the crystallization, the Avrami exponent (*n*) for the D1.4 neat specimen and for the D0.5 neat specimen was *n* = 1.7 for both, suggesting the one-dimensional crystal growth because of the homogenous nucleation for the D1.4 neat and the D1.4/SFN(1.0) specimens in the early stage of the crystallization (*t* < 3.9 min or *t* < 14 min), respectively, for those specimens as reported in our previous paper [11]. However, in the later stage of the crystallization, the Avrami exponent (*n*) was increased from 2.2 (D1.4 neat) to 2.8 (D1.4/SFN(1.0)) by the loading of SFN, which suggests the change of the dimensionality of the crystal growth from 2 dimensional to 3-dimensional by SFN because of the heterogenous nucleation. Such a crossover is also confirmed in the D0.5 neat specimen. If we compare the later stage of the crystallization for the D0.5 neat and D0.5/SFN(1.0) specimens, the Avrami exponent (*n*) is also increased from *n* = 2.2 to 3.0. Such a change of the dimensionality of the crystal growth was observed for the Avrami plots based on the DSC results in our previous study [11] with almost similar values of the Avrami exponent (*n*). These results suggest that the SFN increases the dimensionality of the crystal growth, and hence the crystallization rate (crystallinity increasing rate) is accelerated. 

### 3.2. SAXS Results

**Figure 8:**
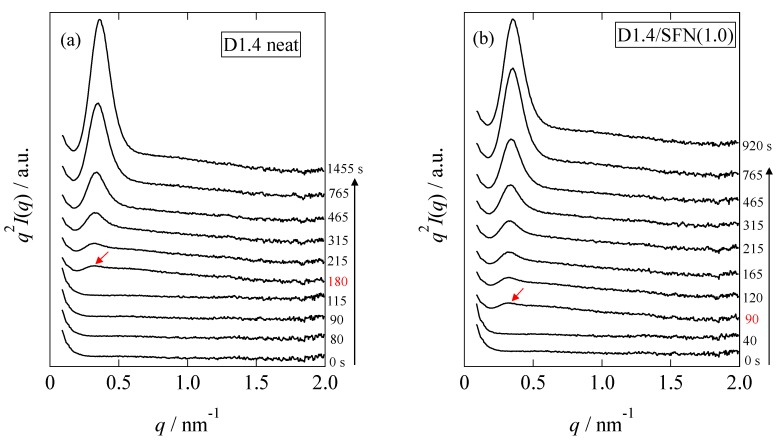
Changes in the Lorentz-corrected SAXS profiles as a function of time for (**a**) D1.4 neat and (**b**) D1.4/SFN(1.0) specimens during the isothermal crystallization at 110 °C from melt. The red arrow indicates the first detection of the peak.

Figure 8 shows the changes in the Lorentz-corrected SAXS profiles as a function of time for the D1.4 neat and D1.4/SFN(1.0) specimens. Here, the scattering intensity, *I*(*q*), is corrected as *q*^2^*I*(*q*) by multiplying *q*^2^. In the early stage of the crystallization, there was no observation of the peak. At *t* = 180 s for the D1.4 neat or *t* = 90 s for the D1.4/SFN(1.0) specimen, a clear scattering peak was observed, which indicates the development of the lamellar stacking with sandwiching the amorphous layers. It is notable to observe that the WAXS peak appears earlier than the SAXS peak (Figure 1 and Figure 8). Such a result indicates that during the early stage of crystallization, the lamellar stacks are incomplete by noting *t*_0_ = 90 s and 40 s for the D1.4 neat and D1.4/SFN(1.0) specimens (see Figure 1). In other words, single lamellae (without stacking) are generated in the early stage of the crystallization.

The intensity of the peak observed at *q* = 0.29 nm^−1^ increases as a function of time. From the peak position (q^*^), the long period (*D*) of the lamellar stacks was evaluated as *D* = 2π/q^*^. As seen in Figure 8, the SAXS peak moves towards the higher *q* as the crystallization proceeds. Increase in *q* suggests the decrease in *D*. 

**Figure 9:**
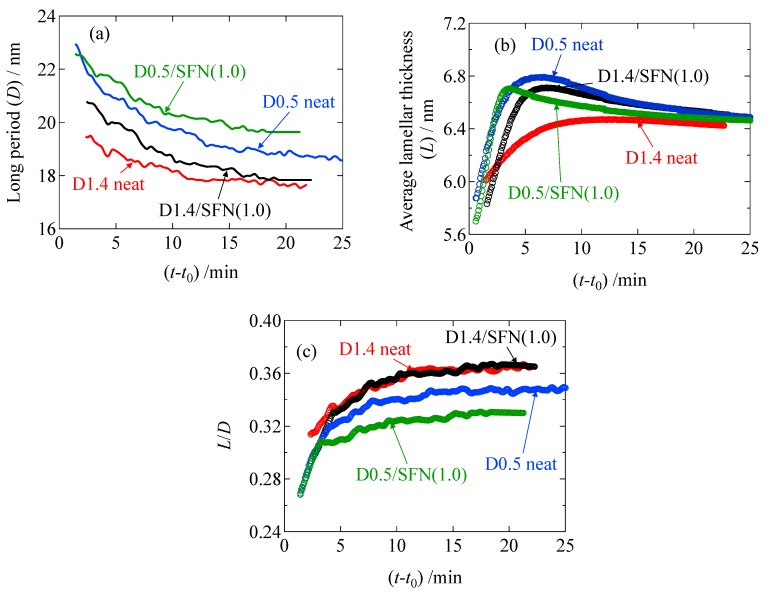
**(a)** Long period (*D*), **(b)** average lamellar thickness (*L*), and **(c)** *L*/*D* as a function of (*t*−*t*_0_), where *t*_0_ is the induction period.

As shown in Figure 9a, the repeating distance of the lamellar stacks (*D*) decreases as a function of the crystallization time. This result seemed to be opposed to the process of crystallization. In order to understand this behavior, we evaluated the average thickness of the crystalline lamella (*L*). To evaluate *L*, the correlation function *γ*(*r*) was calculated from the 1d- SAXS profile through the following equation (inverse Fourier transform method):

(7) $$\gamma \left(r\right)=\frac{\int_{0}^{\infty }I\left(q\right)q^{2}\cos\left(qr\right)dq}{\int_{0}^{\infty }I\left(q\right)q^{2}dq}$$

**Figure 10:**
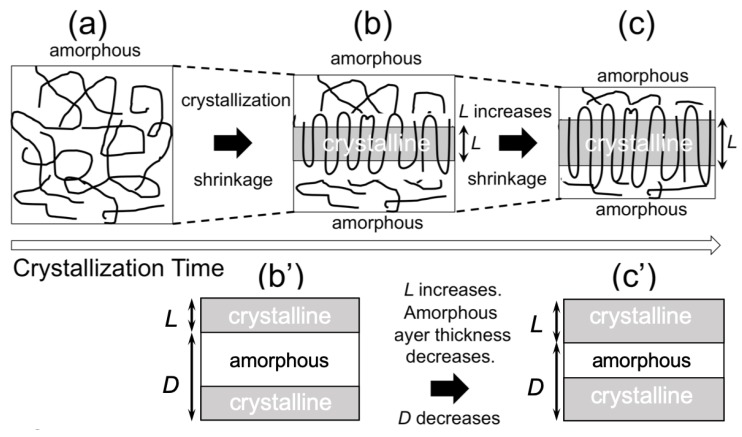
Schematic illustrations showing the change in the nanostructure upon crystallization of poly(l-lactic acid) (PLLA) (**a**) at the amorphous state before crystallization of the polymer melt and (**b**) in an early stage of crystallization. (**c**) Lamellar thickening in the subsequent stage of the crystallization. Note that the illustrations are focusing on the change in the long period (*D*) of the lamellar stacks. **(b’)** and **(c’)** correspond to the states of **(b)** and **(c)**, respectively. The illustrations of **(b’)** and **(c’)** are intended to explain the reason why *D* decreases as a function of time along the proceeding of crystallization where *L* increases with time.

Here, *γ*(*r*) is the correlation function and *r* is the distance in the real space. Figure 9 shows thus-evaluated *L* and *D* and the ratio (*L*/*D*) as a function of time during the isothermal crystallization upon T-jump from 200 °C to 110 °C. As a result (Figure 9b), the average lamellar thickness increases with time, which is reasonable as a crystallization behavior. Therefore, the decreasing behavior of *D* is also reasonable, as schematically shown in Figure 10. Upon crystallization, shrinkage takes place. Since the lamella thickens with time, this results in the decrease of *D* (Figure 10b,c), as an amorphous layer thickness is decreased to a greater extent as compared to the increasing extent of *L* (lamellar thickness). 

It should be noted here that the average lamellar thickness (*L*) did not monotonically increase as a function of time, as the increasing tendency turns over around 7 min for the D0.5 neat specimen or 4 min for the D0.5/SFN(1.0) specimen. The reason why *L* decreased a bit before reaching a constant value may be explained by the formation of new lamellae [17]. It may be explained that in prior to 7 min for the D0.5 specimen or 4 min for the D0.5/SFN(1.0) specimen, a new thin lamellae may be formed from the amorphous region. This argument has been recently reported by our group [18] for the isothermal crystallization of the D0.5 specimen in the presence of a special plasticizer (OMG) at 100 °C from the melt, where a higher order peak was observed in the Lorentz-corrected SAXS profiles.

Furthermore, as shown in Figure 9b and Table 3, the initial average thickness of the lamella (*l*_c_) is thinner for the case of 1% addition of SFN as compared to the neat specimen. This result may explain the mechanism of acceleration of the crystallization by SFN to lower the activation energy of the crystallization with lowering of the thickness of the critical nucleus [11,12]. Note here that *L* = 6.3 nm is the maximum available for the D1.4 sample because of the D moiety (optical impurity), which is distributed with every 70 repeating units of L moieties when the homogeneous distribution of the D moieties in the main chain of PLLA is assumed. Therefore, the final value of *L* in Table 3 for the D1.4 specimens is somewhat larger. This would imply the random distribution of the D moieties in the main chain of PLLA, further meaning that the Gaussian distribution of the L unit repeating length gives its larger value as compared to the average value which results in *L* = 6.3 nm. 

**Figure 11:**
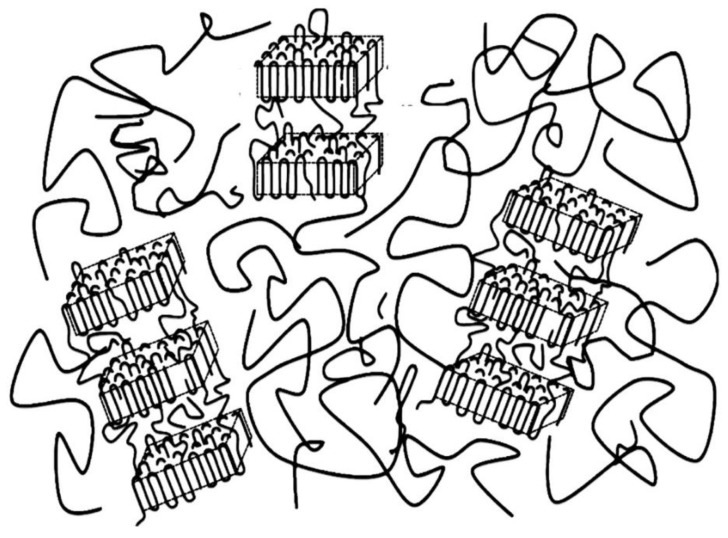
Schematic illustration of the sparsely distributed lamellar stacks in the matrix of polymer melts. This illustrates an early stage of polymer crystallization (adapted from reference [19] with a permission. The figure has been slightly modified).

The ratio *L*/*D* seems to be the crystallinity in the stacks of the lamella. To check the increasing tendency of this ratio as a function of time during the isothermal crystallization, *L*/*D* is plotted as a function of time in Figure 9c. It is then found that the monotonic increase in *L*/*D* is then leveled off without the overshooting, as is seen in the plot of *L* in Figure 9b. Moreover, there is observed no effect of the SFN. This is very much contrasted with the crystallization behavior shown in Figure 2. As shown in Figure 9c, *L*/*D* is larger than *ϕ*_WAXS_ (Figure 2). The reason why *L*/*D* is larger than *ϕ*_WAXS_ is because the lamellar stacks are sparsely dispersed in the matrix of polymer melts, and they do not completely fill the specimen space in the early stage [20]. Such a situation is illustrated in Figure 11. Then, the lamellar stacks completely fill the space in the specimen in the late stage, where the *L*/*D* is almost identical to *ϕ*_WAXS_. It is noteworthy that there is no effect of SFN on the behavior of *L*/*D*, as shown in Figure 9c for the D1.4 neat and D1.4/SFN(1.0) specimens. At this moment, it is unclear that this result is universal, irrespective of the amount of loading. More detailed study is required. As for the D0.5 neat and D0.5/SFN(1.0) specimens, the SFN even reduces the *L*/*D* value, which is opposed to the overall behavior shown in Figure 2. This result may suggest that the SFN enhances the formation of the isolated lamellae without stacking.

## 4. Conclusions

The crystallization behavior of the neat PLLA and PLLA/SFN(1.0) specimens was investigated by using the time-resolved SWAXS techniques. The results showed that the loading SFN shortens the induction period and the increase in the ultimate degree of crystallinity. The crystallization half-time was reduced, which suggests that the overall crystallization process was accelerated. Furthermore, the crystallite size was very slightly reduced, but the growth rate of the crystallites was unchanged. The amount of the α’ and α phases were increased as a function of time, while the fraction was constant (only about 4% of α phase). The dimensionality of the crystal growth was increased due to the inclusion of SFN. The SAXS results suggest that the lamellar thickening process was accelerated, and the thickness of the initial lamella was decreased by loading 1% SFN in PLLA. This result clearly indicates that the thickness of the critical nucleus is reduced by the SFN.

## Figures and Tables

**Table 1:** Sample characterization.

Sample Code	Abbreviation	D Content	Number-Average Molecular Weight (M_n_)	Dispersity (M_w_/M_n_)
PLLA 4032D	D1.4	1.4%	1.66 × 10^5^	2.05
PLLA 2500HP	D0.5	0.5%	1.74 × 10^5^	2.22

**Table 2:** WAXS results for the isothermal crystallization at 110 °C.

Specimens	Induction Period, *t*_0_ (*s*)	Final Degree of Crystallinity, *ϕ*^∞^	Crystallization Half-Time, *t*_0.5_ (min)
D1.4 neat	90	0.22	12.2
D1.4/SFN(1.0)	40	0.27	5.8
D0.5 neat	32	0.25	8.2
D0.5/SFN(1.0)	20	0.31	3.2

**Table 3:** SAXS results for the isothermal crystallization at 110 °C.

Specimens	Initial Average Lamellar Thickness, *l*_c_ (nm)	Final Average Lamellar Thickness, *L* (nm)	Final Long Period (*D*) (nm)	Final Value of *L*/*D*
D1.4 neat	6.01	6.43	17.36	0.37
D1.4/SFN(1.0)	5.83	6.50	17.84	0.37
D0.5 neat	5.88	6.49	18.60	0.35
D0.5/SFN(1.0)	5.70	6.48	19.64	0.33
